# Emphysematous cystitis, iliopsoas abscess, and pneumorrhachis in an elderly woman: a case report

**DOI:** 10.1186/s13256-023-03856-7

**Published:** 2023-04-10

**Authors:** Maya Stein, Adam Min, Basma Mohammed, Shobhit Mathur, Jonathan Ailon

**Affiliations:** 1 Department of Internal Medicine, St. Michael’s Hospital, University of Toronto, Toronto, ON Canada; 2 Department of Radiology, St. Michael’s Hospital, University of Toronto, Toronto, ON Canada

**Keywords:** Case report, Emphysematous cystitis, Pneumorrhachis, Iliopsoas abscess, Gangrene, Sepsis

## Abstract

**Background:**

Emphysematous cystitis is a well-described life threatening complication of urinary tract infection, most commonly seen in patients with diabetes and typically caused by gas forming bacterial or fungal pathogens. Pneumorrhachis is the rare finding of gas within the spinal canal, most commonly reported in the context of cerebrospinal fluid leakage secondary to trauma or spinal instrumentation. To our knowledge there is only one other reported case of pneumorrhachis in the setting of emphysematous cystitis.

**Case presentation:**

This is a single case report of pneumorrhachis in the setting of emphysematous cystitis. An 82-year-old Asian female patient originally from East Asia, with no prior medical history besides hypertension, presented to hospital with a chief complaint of acute on chronic neck pain and functional decline. Examination revealed nonspecific neurosensory deficits and suprapubic tenderness. Laboratory investigations demonstrated leukocytosis and extended-spectrum beta-lactamase containing *Escherichia coli* bacteremia and bacteriuria. Computed tomography showed emphysematous cystitis with widespread gas within the cervical and lumbar spinal canal, as well as multiple gas-containing soft tissue collections in the bilateral psoas muscles and paraspinal soft tissues. Despite prompt antimicrobial therapy the patient passed away within 48 hours from septic shock.

**Conclusions:**

Our case adds to a growing body of literature showing that the spread of air to distant sites, including the spine, may be a poor prognostic indicator in patients with gangrenous intraabdominal infections. This report highlights the importance of recognizing the causes and presentation of pneumorrhachis to facilitate early diagnosis and treatment of potentially life threatening and treatable causes.

## Introduction

Emphysematous cystitis is a life threatening, necrotizing infection of the bladder characterized by air within the bladder wall. It is most commonly seen in patients with diabetes and is caused by gas forming bacterial or fungal infections [1]. Pneumorrhachis is the rare finding of gas within the spinal canal. Most commonly, pneumorrhachis is due to a cerebrospinal fluid (CSF) leak secondary to trauma or spinal instrumentation; however, it can occur rarely in the context of gangrenous intraabdominal infection [2]. We describe a case of an elderly female patient who presented with concurrent emphysematous cystitis, iliopsoas, and paraspinal abscesses, and pneumorrhachis. We present theories regarding the origin and spread of air to distant sites, such as the spine, in our patient.

## Case presentation

An 82-year-old Asian female presented to the emergency department with acute on chronic neck pain, weakness, and functional decline. Two months prior to her presentation, she developed worsening neck pain and lower extremity weakness, which quickly progressed to near paralysis, resulting in her becoming bed-bound. Her neck pain was described as a posterior sharp, stabbing-like pain, which progressed in intensity over 2 months and limited her ability to mobilize in the week preceding presentation. She also reported paresthesias and numbness in her lower extremities bilaterally. She denied a history of fevers, chills, or other focal neurologic deficits such as photosensitivity or headaches. She also reported intermittent lower abdominal pain and discomfort over the preceding month, but denied further genitourinary symptoms such as dysuria or urinary frequency. Prior to the onset of these symptoms, she had been entirely functionally independent and previously worked as a pharmacist. The patient’s medical history was notable only for hypertension. She had no recent history of trauma or injury. She had no history of diabetes mellitus. Her only medication was an angiotensin receptor blocker. The patient had immigrated to Canada from East Asia approximately ten years prior to this presentation and had minimal contact with the healthcare system since immigrating to Canada. She was a lifetime nonsmoker and did not consume alcohol or illicit drugs.

## Clinical findings

On initial assessment, the patient appeared distressed and diaphoretic. Her temperature was 37.4 °C. The remainder of the vital signs were within normal ranges. Axial rotation of the neck was limited to 20° left and right due to pain. There was marked tenderness to palpation along the cervical spine. She also had weakness, with 1/5 power on flexion and extension of the lower extremities bilaterally. Plantar reflexes were up-going bilaterally, and there was diminished sensation to light touch bilaterally, below L2 dermatome. Upper extremity strength was normal. Jolt accentuation, Kernig, and Brudzinski signs were all negative. Abdominal examination revealed suprapubic tenderness, without guarding, rebound tenderness, or rigidity. The remainder of the physical examination was normal.

## Diagnostic assessment and interventions

**Fig. 1:**
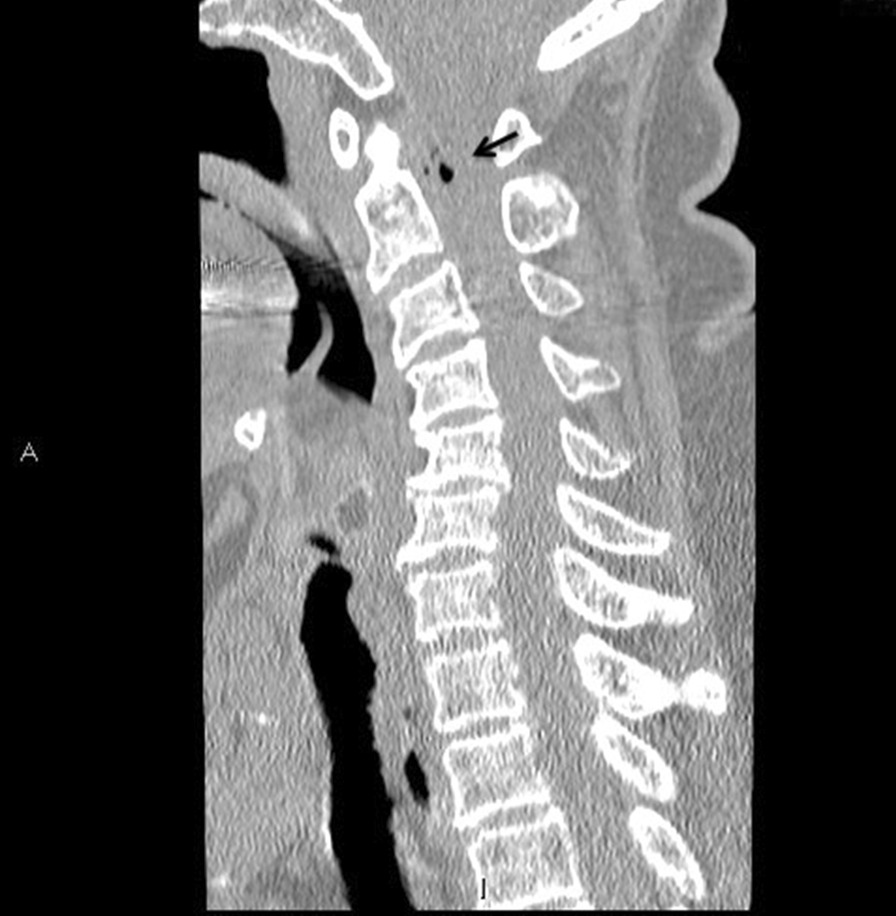
Computed tomography of the cervical spine demonstrating locules of gas within the upper cervical spinal canal (black arrow)

**Fig. 2:**
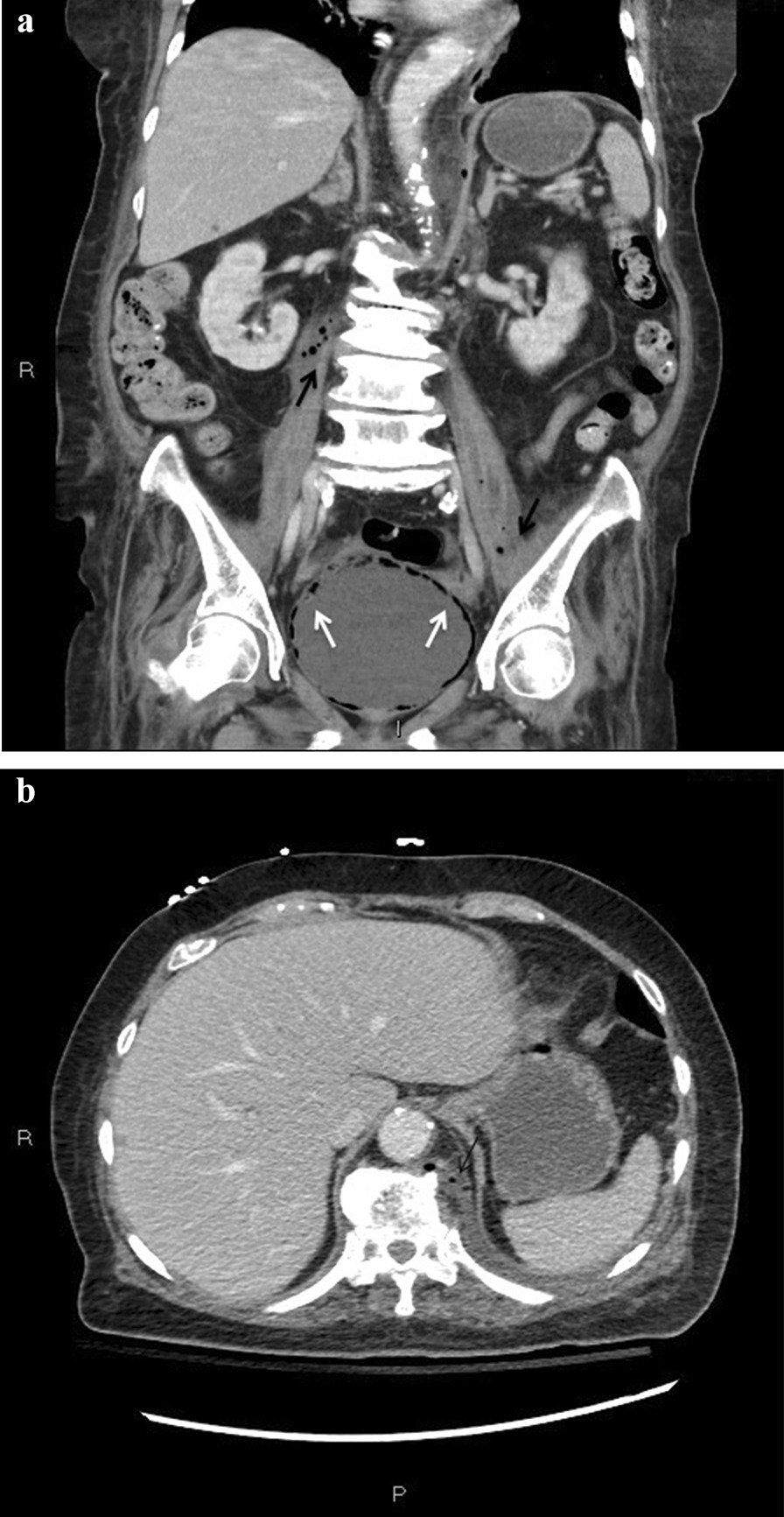
**a** Computed tomography of the abdomen and pelvis demonstrating extensive gas within the bladder wall (white arrows), consistent with emphysematous cystitis, as well as bilateral gas-containing psoas abscesses (black arrows). **b** Computed tomography of the abdomen and pelvis demonstrating a gas-containing paraspinal collection (black arrow)

**Fig. 3:**
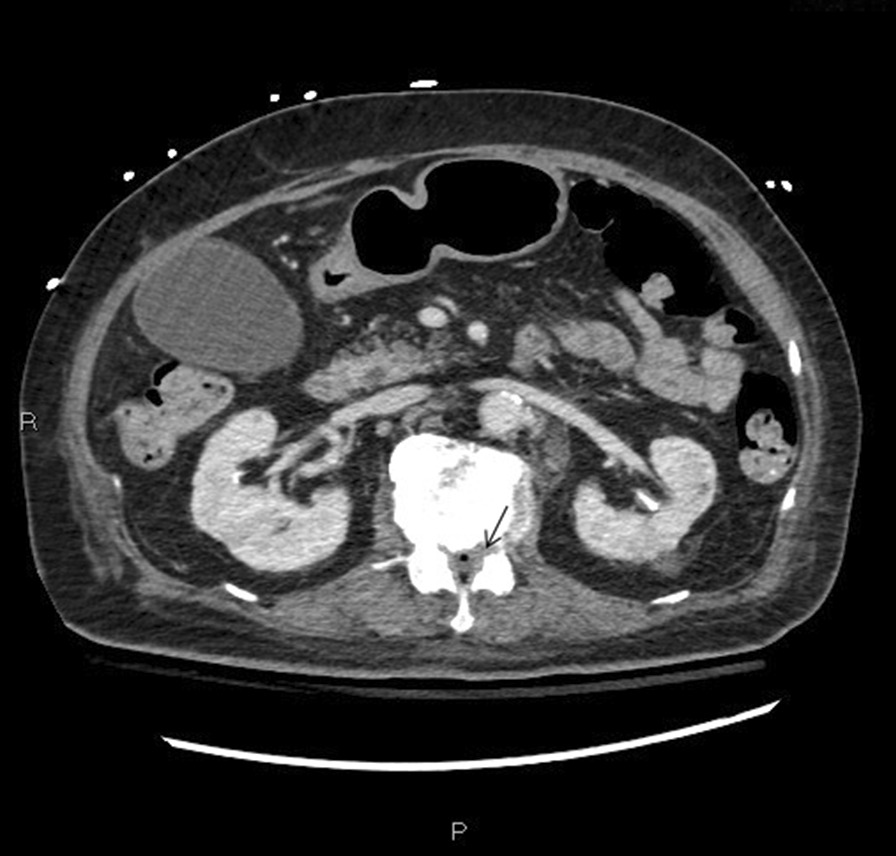
Computed tomography of the abdomen and pelvis demonstrating gas within the lumbar spinal canal (black arrow)

**Fig. 4:**
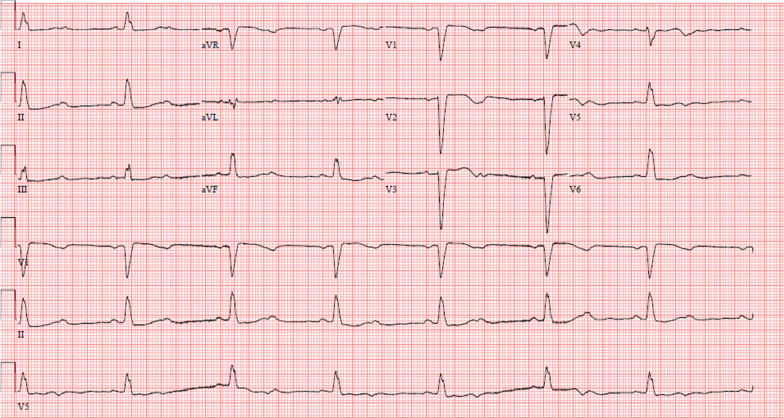
Electrocardiogram demonstrating complete heart block

**Fig. 5:**
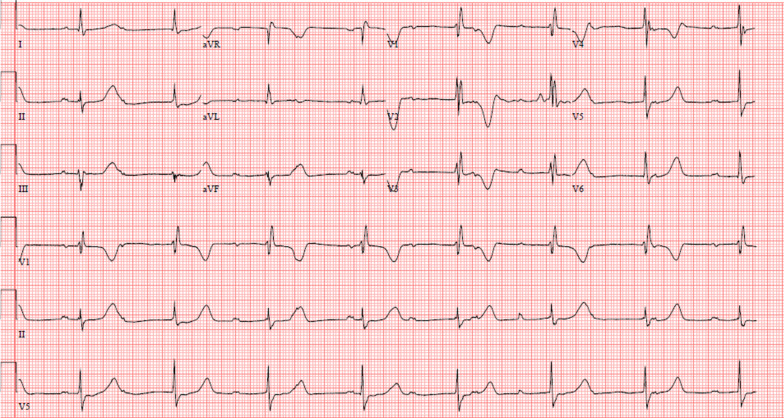
Initial electrocardiogram on admission demonstrating second degree atrioventricular block with 2:1 conduction

**Fig. 6:**
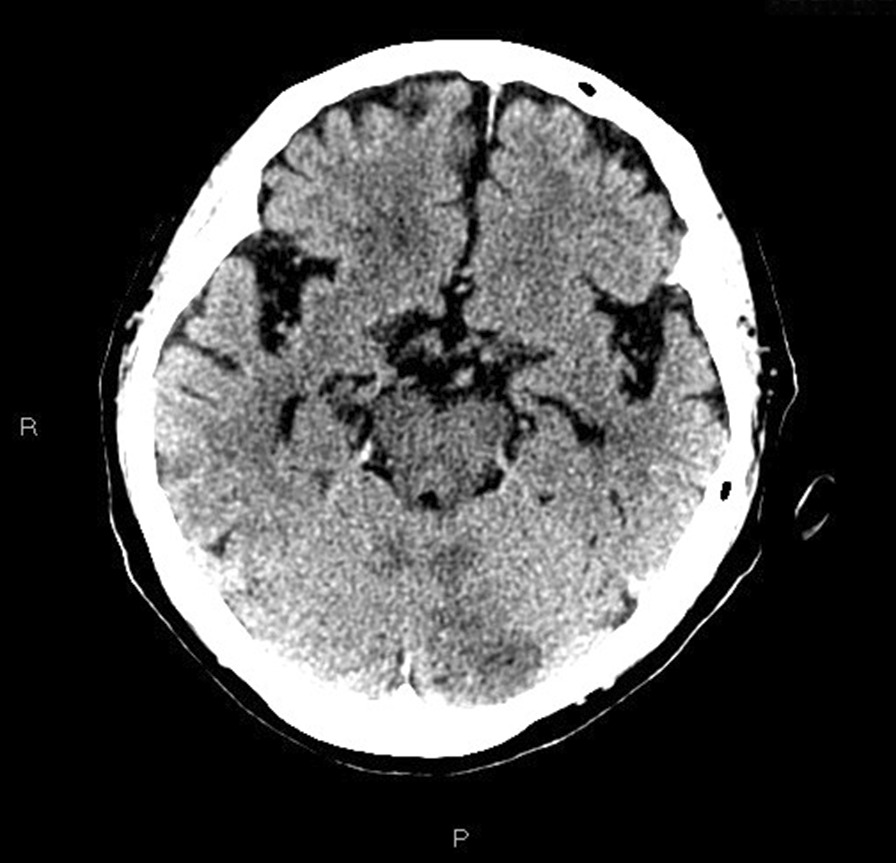
Non-contrast head computed tomography demonstrating a subacute infarct in the left cerebellar hemisphere

Initial laboratory investigations revealed leukocytosis of 17.3 × 10^3^/L with neutrophil predominance and lactate of 4.7 mmol/L, platelet count of 44 × 10^9^/L, and urinalysis showing 3+ proteinuria with positive nitrites and leukocytes. Other laboratory test results are shown in Table 1. An initial computed tomography (CT) of the head and cervical spine (Fig. 1) demonstrated a locule of gas within the upper cervical spinal canal, without a clear source. Further investigation with CT of the thorax, abdomen, and pelvis was performed (Fig. 2), which demonstrated multiple gas-containing soft tissue abscesses within the bilateral psoas muscles and thoracic paraspinal soft tissues, as well as emphysematous cystitis. Additional locules of gas were also demonstrated within the lumbar spinal canal (Fig. 3). There was no evidence of emphysematous pyelonephritis. Overall, the constellation of findings raised concern for widespread infection with a gas-forming organism. Blood and urine cultures returned positive for Gram-negative bacilli, and the patient was treated empirically with ceftriaxone 2 g intravenously daily. Over the next 24 hours, the patient rapidly deteriorated. Her blood and urine cultures speciated to extended-spectrum beta-lactamase (ESBL) positive *Escherichia coli* and her antibiotics were broadened to meropenem 500 mg every 6 hours. She subsequently became hemodynamically unstable with hypotension and bradycardia, with a heart rate in the 30s (beats per minute). An electrocardiogram (ECG) (Fig. 4) demonstrated new high-grade atrioventricular (AV) block, prompting transfer of the patient to the cardiac critical care unit for cardiac pacing and advanced supportive care. Of note, her initial ECG on admission (Fig. 5) demonstrated second degree AV block with 2:1 conduction; there was no prior ECG for comparison. Within the next 12 hours, the patient developed sudden onset slurred speech and decreasing level of consciousness. A code stroke protocol CT demonstrated new bilateral cerebellar infarcts, with proximal basilar artery occlusion (Fig. 6). The source of her brainstem stroke was presumed to be from critical illness and an existing clot, which was either embolic or thrombosis *in situ* due to preexisting atherosclerotic plaque. There was no evidence of vegetation on a transthoracic echocardiogram, and no suggestion of disseminated intravascular coagulation (DIC) on review of peripheral smear. She was not a candidate for endovascular treatment or tissue plasminogen activator. She was intubated for airway protection. Over the subsequent hours the patient developed refractory hypotension, despite vasopressive medications, owing to her ongoing septic shock, and her heart rhythm progressed to asystole.

**Table 1:** Laboratory investigations

Variable	Reference ranges for hospital	On presentation	48 hours after presentation
WBC (× 10^9^/L)	4.00–11.00	17.30	19.02
RBC (× 10^12^/L)	3.80–5.20	4.05	3.55
HGB (g/L)	115–155	128	110
HCT (L/L)	0.370–0.480	0.370	0.348
PLT (× 10^9^/L)	140–400	44	148
Abs. neutrophils (× 10^9^/L)	2–6.3	14.64	14.64
Abs. lymphocytes (× 10^9^/L)	1–3.2	1.49	2.48
pH venous	7.35–7.42	7.42	7.57
H ion venous (mmol/L)	38–45	30	27
pCO_2_ venous (mmHg)	42–52	30	22
Bicarbonate venous (mmol/L)	21	25	21
Lactate (mmol/L)	0.5–2.3	2.5	4.9
Troponin (ng/L)	< 18	17	46

*WBC* White blood cell count, *RBC* Red blood cell count, *HGB* Hemoglobin, *HCT* Hematocrit, *PLT* Platelets, *Abs. neutrophils* Absolute neutrophils

## Discussion

There are simultaneously two rare processes driving this patient’s presentation: pneumorrhachis and emphysematous cystitis. Pneumorrhachis is the finding of intraspinal gas, which can be intradural, extradural, or both [3]. Most commonly, pneumorrhachis is post-traumatic or iatrogenic in nature, but has also been reported in cases of intraspinal infection, gas-containing synovial cysts, and as a complication of intravenous drug abuse [2]. Rarely, pneumorrhachis has been described in the setting of a gangrenous intraabdominal infection, as in our case. To our knowledge, there has only been one other reported case of pneumorrhachis in the setting of emphysematous cystitis [4]. Three other case reports describe emphysematous pyelonephritis and pneumorrhachis [2, 5, 6]. Emphysematous cystitis is the finding of free air in the bladder wall and lumen [1], typically a consequence of untreated complicated urinary tract infection, especially in elderly women with diabetes mellitus. Mortality rates have been reported to be as high as 90% in medically managed patients [7], owing to the severity and rapidity of infection spread with gas-producing organisms. Management of emphysematous cystitis consists of prompt antimicrobial therapy, urinary catheterization, glycemic control, and in some cases, percutaneous laparoscopic or even open surgical intervention. Management of pneumorrhachis is largely aimed at treating the underlying cause, but may require surgical intervention for complications such as tension pneumorrhachis with spinal cord compression [8]. The clinical presentation for both emphysematous cystitis and pneumorrhachis is nonspecific, and a high index of clinical suspicion and radiographic confirmation are required to make the diagnosis. While patients with emphysematous cystitis may present with symptoms of lower urinary tract infection including dysuria, suprapubic pain, and fever, many are diagnosed on imaging alone, without clinical symptoms [9]. Pneumorrhachis is usually asymptomatic but can present with pain and discomfort in conjunction with nonspecific neurologic sensorimotor deficits, as in our patient [3, 10].

Both emphysematous cystitis and pneumorrhachis are inherently image-dependent diagnoses, requiring the visualization of air within the bladder wall and thecal sac, respectively. Most commonly, they are diagnosed on CT or ultrasound, often performed to rule out other causes of the patient’s symptoms [5]. Magnetic resonance tomography (MRT) may be a helpful adjunct investigation, especially in evaluating for spondylodiscitis or spinal and soft tissue abscesses.

There are several theories regarding the origin and route of infection and the route of spread in our patient. The first possibility, which we favor, is that the infection began within the bladder, and subsequently tracked into the bilateral iliopsoas muscles, paraspinal muscles, and eventually traversed into the spinal canal. There was a high likelihood the patient also had discitis and/or epidural abscess, given the distribution of disease; however, this was not confirmed as the patient was too medically unstable to safely perform MRT of the spine. The second theory is that the disease process began within the spine and subsequently proceeded to the bladder. Lastly, given that the patient was bacteremic, hematologic dissemination of infection is another possibility. Nevertheless, determining the precise route of infection would not have impacted the management or the outcome of the patient.

## Conclusions

Our case adds to the growing body of literature describing pneumorrhachis in the setting of emphysematous intraabdominal infections. In our patient, pneumorrhachis presented with a chief complaint of neck pain and was discovered incidentally on a noncontrast CT of the cervical spine, prompting further investigation and leading to the diagnosis of emphysematous cystitis. This highlights neck pain as an important presenting clinical symptom of pneumorrhachis. Given the rapidity of disease progression in our patient, we argue that the spread of air to distant sites, including the spine, should be considered a poor prognostic indicator in gangrenous intraabdominal conditions, such as emphysematous cystitis.

## Data Availability

All data acquired for this case report are included in the submitted article and its additional information files.

## Abbreviations

CSF: Cerebrospinal fluid
CT: Computed tomography
ECG: Electrocardiogram
MRI: Magnetic resonance imaging
ESBL: Extended-spectrum beta-lactamase
DIC: Disseminated intravascular coagulation
AV: Atrioventricular
